# Investigating the Protective Effect of Gross Saponins of *Tribulus terrestris* Fruit against Ischemic Stroke in Rat Using Metabolomics and Network Pharmacology

**DOI:** 10.3390/metabo9100240

**Published:** 2019-10-21

**Authors:** Yang Wang, Wenjun Guo, Yue Liu, Jifeng Wang, Meiling Fan, Hongyu Zhao, Shengxu Xie, Yajuan Xu

**Affiliations:** 1 Jilin Ginseng Academy, Changchun University of Chinese Medicine, Changchun 130117, China; wangyang@ccucm.edu.cn; 2 School of Pharmaceutical Sciences, Changchun University of Chinese Medicine, Changchun 130117, China; 19969540912@163.com (W.G.); wjf02166@163.com (J.W.); 3 Key Laboratory of Medicinal Materials, Jilin Academy of Chinese Medicine Sciences, Changchun 130021, China; yue_liu_tryh@126.com (Y.L.); fanmeiling1982@163.com (M.F.); fixiov5815@126.com (H.Z.); jshx222@126.com (S.X.)

**Keywords:** ischemic stroke, gross saponins of *Tribulus terrestris*, LC-MS-based metabolomics, network pharmacology

## Abstract

Stroke is one of the leading causes of death and long-term disability worldwide. Gross saponins of *Tribulus terrestris* fruit (GSTTF) has been used for neuroprotective therapy on convalescents of ischemic stroke. But the related therapeutic mechanisms have not yet been well investigated. This study aimed to investigate the protective effects of GSTTF on ischemic stroke using metabolomics coupled with network pharmacology analysis. The rat urine sample was collected and profiled by an LC-MS-based metabolomics approach. The pathway analysis was performed based on the highlighted biomarkers, then the network pharmacology approach was applied to screen the potential therapeutic targets of GSTTF. Metabolomics analysis showed that a series of metabolic perturbations occurred in the middle cerebral artery occlusion (MCAO) group compared with the sham group. Gross saponins of *Tribulus terrestris* fruit can change the MCAO-induced urine metabolic deviations in a reverse manner via regulating multiple metabolic pathways. Two proteins, inducible nitric oxide synthase (NOS2) and glycogen synthase kinase-3 beta (GSK3B), were highlighted by the network pharmacology analysis, which may be the potential therapeutic targets for the GSTTF against ischemic stroke. This study provides an overview of the mechanism of MCAO-induced ischemic stroke and investigates the efficacy of GSTTF in the treatment of ischemic stroke. Further study is needed to reveal its underlying mechanisms more clearly.

## 1. Introduction

Stroke is one of the leading causes of death and long-term disability worldwide. The origin of over 80% of all strokes are ischemic events, which can result in harmful neurological sequelae accompanied by severe morphological and molecular alterations [1,2]. As stated by the World Health Organization (https://www.who.int/topics/cerebrovascular_accident/en/), ischemic stroke is chiefly caused by the interruption of the blood supply to the brain when a blood vessel bursts or is blocked by a clot, which can cut off the supply of oxygen and nutrients thus bringing damage to the brain tissue. Currently, thrombolysis is considered as the first-choice treatment for ischemic stroke, which tries to dissolve the clots, restore blood flow, and protect the surrounding brain tissue. The mechanisms and methods for a precise pathology of ischemic brain injury have not yet matured; there are no generally accepted and effective treatments for ischemic stroke to date [3,4].

Traditional Chinese herbal medicine (TCHM) has shown unique therapeutic effects for various diseases and disorders with the advantages of little side effects and low cost. Several traditional Chinese medecines (TCMs), including Danqi Piantan capsules, ginkgolides injection, and Buchang Naoxintong capsules, have been investigated for their protective effect and treatment toward cerebral ischemia [5,6,7]. *Tribulus terrestris* L. is an annual creeping herb widespread in China, India, USA, Mexico, and Spain. Jili is the fruit of *Tribulus terrestris* that has been used to treat eye disease, edema, abdominal distention, sexual dysfunction, high blood pressure, and cardiovascular diseases [8,9,10]. The gross saponins of Jili (GSTTF) are a mixture of dozens of different types of saponins, mainly steroid saponins, which have been reported to protect the brain from ischemic injury in vivo and in vitro [11,12]. However, few studies have applied the metabolomics technique to investigate the mechanisms of its protective effect [13]. 

Metabolomics is a comprehensive technique to profile entire endogenous metabolites in the living system, which could profile the global changes of end products in biological samples, and has been widely applied in biomarker discovery, toxicity evaluation, and drug efficacy assessments. According to the holistic philosophy of TCM, metabolomics has great potential to integrate ancient TCM with modern medicine, and, furthermore, facilitate the modernization of TCM [14,15]. Network pharmacology is developed based on the increasing knowledge of the network of genes and molecular interactions, which has been considered as a holistic and efficient technique to underly the disease pathogenesis and the therapeutic mechanisms of TCM [16,17].

The present study aims to evaluate the protective effect of GSTTF against ischemic stroke in rat. A middle cerebral artery occlusion (MCAO) model was constructed and injected with GSTTF via tail vein injection. The changes in urine metabolite were monitored by liquid chromatography-mass spectrometry (LC-MS)-based metabolomics to select the urine biomarkers and analyze the perturbed metabolic pathway associated with MCAO. Then, the protective effect of GSTTF during the pathogenesis of ischemic stroke was further investigated by the network pharmacology technique. It was expected to provide a reference to understand the pathogenesis of ischemic stroke and the therapeutic mechanisms of GSTTF.

## 2. Results

### 2.1. The Effects of GSTTF on MCAO in Rats

**Figure 1:**
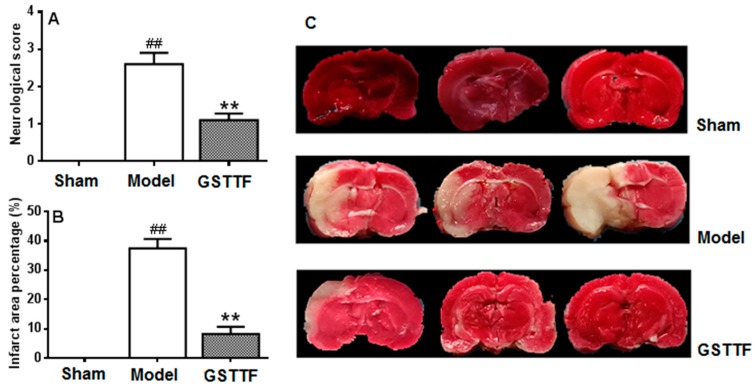
Effects of Gross saponins of *Tribulus terrestris* fruit (GSTTF) on middle cerebral artery occlusion (MCAO) rats: neurobehavioral score (**A**), infarct area (**B**), and 2,3,5-triphenyltetrazolium chloride (TTC) staining of brain (**C**). ^##^ *p* < 0.01, the model group versus the sham group; ** *p* < 0.01, the GSTTF-treated group versus the model group.

Neurologic score and infarct volume measurement were conducted to evaluate the animal model and the effect of GSTTF for ischemic stroke. As shown in Figure 1A, the rat from the sham group did not have any neurological defect and had a score of zero, while the rats in the model group showed the highest score among the three groups, suggesting a neurological defect after MCAO. After treated with GSTTF, the neurological defect was significantly reversed compared to the model group.

The infarct volume of each group was shown in Figure 1B,C, no infarct area was observed in the brain tissue from the rats in the sham group. A remarkable infarct area in the coronal brain sections was yielded after MCAO surgery, which is consistent with their highest neurological defect score among three groups. A significant recovery in the infarct area was observed in TTC-stained cerebral slices after treated with GSTTF. The results indicate that the administration of GSTTF prior to MCAO had a neuroprotective effect in rats.

### 2.2. Metabolic Profile in Urine

**Figure 2:**
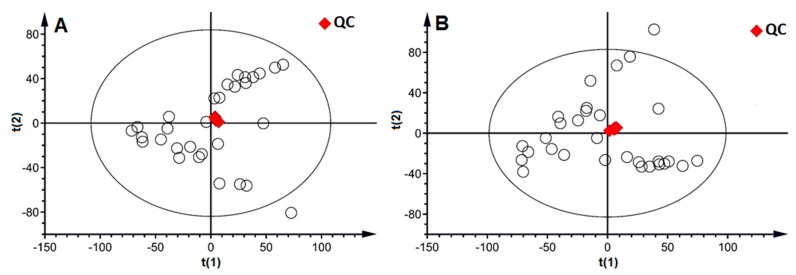
The PCA score plot of all samples (○) with the highlight of the QC (♦) sample in positive ion mode (**A**) and negative ion mode (**B**).

The reproducibility and stability of the LC-MS method were fundmental in the metabolomics analysis and were assessed by using the quality control (QC) samples. Five injections of QC samples were running for system conditioning initially, and then one injection was analyzed every fourth analytical run to provide a dataset for the assessment of repeatability and stability. A total of 8413 and 7879 peaks were extracted from the urine sample in positive ion mode and negative ion mode, respectively. After alignment and normalization, the dataset from all samples was used to perform principal component analysis (PCA). As shown in the PCA score plot (Figure 2), the relatively tight clustering of the QC samples at the center of all samples was observed in both positive and negative ion modes, suggesting the good repeatability and stability of the developed method. 

The urine metabolite was profiled by the validated UHPLC-Q-Orbitrap/MS method, and the typical base peak chromatograms (BPC) of three groups detected in both positive and negative ion modes are illustrated in Supplementary Materials Figure S1. Good separation was accomplished in 12 min by the established method, and several differences among these groups were observed, e.g., the peak at 2–4 min. Then, the multivariate statistical analysis was conducted to investigate the changes in urine metabolite in the three groups and further to evaluate the protective effect of GSTTF. 

**Figure 3:**
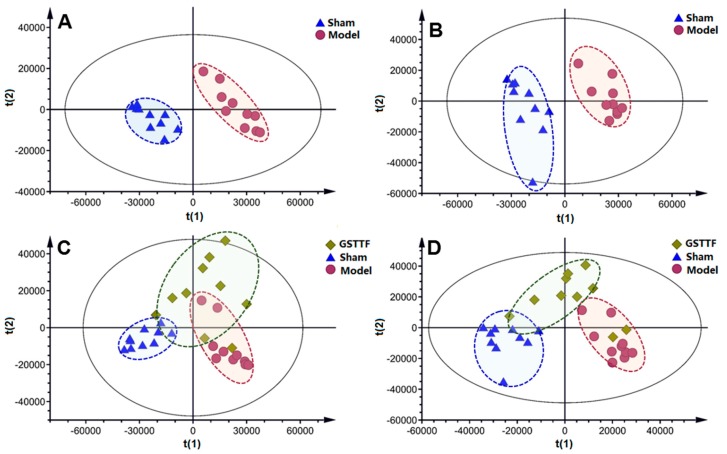
The PLS-DA score plots of the sham group (▲), model group (●), and GSTTF-treated group (♦) based on the data acquired in positive ion mode (**A**,**C**) and negative ion mode (**B**,**D**).

A partial least squares discriminant analysis (PLS-DA) model was constructed to offer an outline of the differences in urine metabolic profiling between the sham group and the model group. The parameters R2Y and Q2 value of 0.997 and 0.938 in positive ion mode and 0.995 and 0.930 in negative ion mode were considered to be an excellent fitness and prediction ability of the established PLS-DA model. The results of the permutation test (*n* = 200) in Figure S2 showed that the value of intercepts to the left was lower than the original value, suggesting the great predictability and goodness of fit of the established model. As shown in the score plot of PLS-DA (Figure 3A,B), clear separation among these two groups was observed in the first principal component, suggesting the urine metabolome was altered by the MCAO surgery. The GSTTF-treated group showed a trend close to the sham group and away from the model group (Figure 3C,D), which indicates that after being treated with GSTTF, the metabolic changes trend in the control group, and the GSTTF had a certain protective effect on ischemic stroke.

### 2.3. Biomarker Selection and Metabolic Pathway Analysis

Multiple criteria were applied, including variable importance in the projection (VIP) value, fold change, and *p*-value, to select the metabolites that contributed most to the classification of control group and model group. Specifically, variables with VIP1 and VIP2 values (>2.0) were highlighted as candidate biomarkers, and the variables were further screened by the fold change and student’s *t*-test to select the features with a significant difference among two groups. The metabolite with a fold change > 2 and *p* < 0.05 were highlighted as the potential biomarkers. The structure of these compounds was tentatively assigned by comparing the MS and MS/MS spectra with the detected metabolites by searching the Human Metabolome Database (HMDB) [18] and METLIN databases [19]. The hippuric acid ion at RT 3.99_*m*/*z* 178.0502 in negative ion mode was used as an example to demonstrate the annotation procedure. As shown in Figure S3, the ion at *m*/*z* 178.0503 in MS spectrum corresponding to the [M − H]^−^ of hippuric acid, and the ion at *m*/*z* 160.0398 and 134.0601 in MS/MS spectrum represent the fragment ion of [M − H_2_O]^−^ and [M − HCOOH]^−^, respectively. Finally, 14 potential biomarkers related to the MCAO were tentatively assigned and are shown in Table 1. These metabolite changes in urine reflected the alterations in the metabolic phenotype, which could provide insight into the underlying mechanism of stroke.

**Figure 4:**
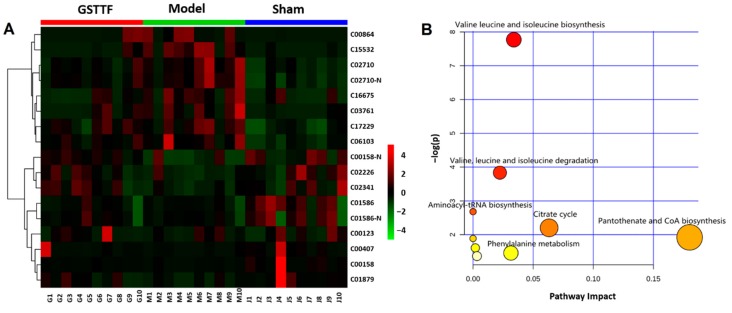
Heat map (**A**) visualizing the changes in the intensities of potential biomarkers and bubble plot (**B**) of the main perturbed pathway response to MCAO.

A heat map was constructed using the MetaboAnalyst software [20] based on the intensity of each biomarker to display the overview of the metabolites’ changes in three groups. As depicted in Figure 4A, color differences between model group and sham group indicate the metabolic perturbation after MCAO surgery. Compared to the sham group, the intensities of these metabolites changed in the model group, while in the GSTTF-treated group, the level of these metabolites partly recovered to close to the level in the sham group. 

To explore the possible pathways related to the MCAO, the biomarkers were input into the MetaboAnalyst software for pathway analysis and visualization. As shown in Figure 4B, the significant relevant pathways influenced by MCAO surgery, including valine leucine and isoleucine biosynthesis, citrate cycle, pantothenate and CoA biosynthesis, beta-alanine metabolism, glutathione metabolism, etc., are highlighted as the most important metabolic pathways. 

### 2.4. Network Pharmacology Analysis

As shown in Figure S4A, the compound–target (C-T) network for the active compound of *Tribulus terrestris* and the targets it regulates were constructed with 142 nodes (27 active compounds and 115 candidate targets) and 267 edges. This complex network shows all the targets that the compounds can modulate. Figure S4B shows the disease–target (D-T) network, which connects the encephalopathy-related diseases, including Alzheimer’s disease, brain injury, cardiovascular disease, inflammation, ischemic stroke, and thrombosis, and their therapeutic targets. A total of 25 disease-related targets were obtained from this network.

**Figure 5:**
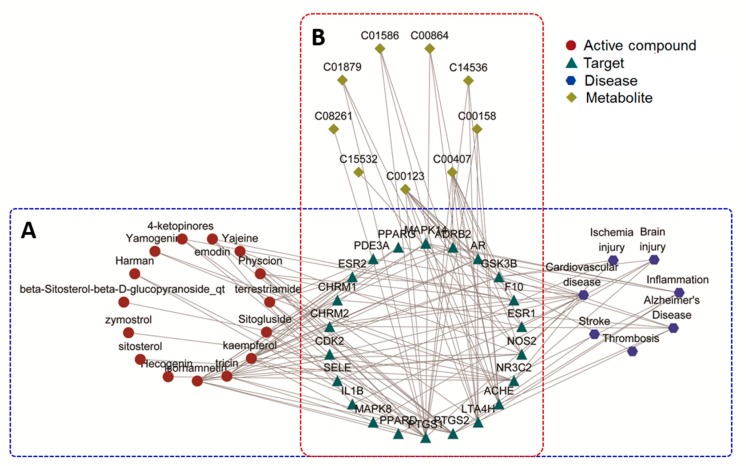
The “compound-target-disease” network (**A**) and “target-metabolite” network (**B**) based on 15 compounds, 22 targets, 7 diseases, and 9 metabolites.

To further clarify the relationships between the active compound and the disease, the active compound–target-disease network (C-T-D) network was constructed by merging the above two networks. As shown in Figure 5A, the C-T-D network consists of 44 nodes (15 active compounds, 22 targets, and 7 diseases) and 127 edges. Twenty-two targets (Table S1) that can be modulated by 15 active compounds and related to the disease were selected from the C-T network and D-T network; these may be the potential therapeutic target of *Tribulus terrestris* for encephalopathy. Then, these targets and biomarkers selected from the metabolomics analysis were further input into the IMPaLA web tool to perform a joint pathway analysis [21]. As a result, shown in Figure 5B, nine targets displayed a connection with the nine metabolites that associated with MCAO, from which two targets were correlated with ischemic stroke, suggesting that these two targets were probably the potential target for the protective effect of GSTTF against the MCAO.

## 3. Discussion

Urine samples from the model group, sham group, and GSTTF-treated group were analyzed by LC-MS to explore the metabolite change among the three groups and to evaluate the protective effect as well as the associated mechanism of GSTTF. The result of infarct volume analysis and neurological defects evaluation suggested the protective effect of GSTTF for ischemic stroke. In agreement with this result, the PLS-DA score plot showed that the cluster of the GSTTF-treated group was separated from the model group and trended towards the sham group, but still had some overlap with the model group. Previous serum metabolomics analysis showed that the GSTTF-treated group was totally separated from the model group and close to the sham group [13]; compared with that, the present study suggests that the metabolites in urine were less affected by GSTTF, but still have a trend toward to the sham group. A total of 14 metabolites were selected as biomarkers and their related pathways were further investigated. Network pharmacology analysis highlighted two potential targets that could be considered as the protective effect of GSTTF for ischemic stroke. 

Our previous study using gas chromatography (GC)-MS-based metabolomics to analyze serum metabolome showed that the MCAO surgery significantly influence the fatty acid metabolism, amino acid metabolism, carbohydrate metabolism, etc. [13]. In this study, the metabolites involved in valine leucine and isoleucine metabolism, citrate cycle, and phenylalanine metabolism, etc., were screened as biomarkers of MCAO. 

Leucine and isoleucine, two branched-chain amino acids (BCAAs), were greatly decreased in the model groups compared with that in the sham group, which is consistent with the previous reports using the targeted method [22] and GC-MS-based untargeted method [23]. Branched-chain amino acids have been proven to play a vital role in the metabolic response of several diseases, such as cardiovascular diseases, type 2 diabetes, and insulin resistance [24,25,26]. Many biological processes can be regulated by BCAAs, including anabolism (protein synthesis), catabolism (energy production), de novo synthesis of glutamate, as well as influence the transport of tryptophan, tyrosine, and phenylalanine across the blood–brain barrier. Differential changes in circulating BCAAs may be connected with the nutritional status of ischemic stroke subjects responding to anabolic and catabolic changes or metabolic comorbidities [27,28]. Isoleucine and leucine are important energy sources of the citrate cycle after being converted to acetyl-CoA for energy supply. The reduction of these two BCAAs might be caused by their utilization as cell signaling molecules or consumption of citric acid for reactivation of brain function. 

The decreased level of citric acid was observed in the MCAO group, indicating that brain injury of in rats can downregulate the citrate cycle. Stroke can induce the inhibition of the citrate cycle and further reduce ATP production, which lead to the utilization of BCAAs as energy compensation; thus, these amino acids are of great significance in bioenergetic homeostasis. Citric acid is an important intermediate in the citrate cycle and was reported to be reduced in stroke subjects. Published metabolomics studies using urine and blood samples have also reported that stroke patients had relatively lower levels of citric acid [29,30], which was in agreement with our results. This consistent set of findings suggests that stroke is associated with inhibition of the citrate cycle. 

Phenylalanine metabolism was also altered from the pathway analysis. Hippuric acid is one of phenylalanine’s metabolites, and a significant decrease in this metabolite was observed in the model group in this study which could be caused by the reduced consumption of phenylalanine. The previous study showed that the level of phenylalanine considerably increased in stroke subjects compared to healthy controls, indicating the phenylalanine metabolism pathway is altered in stroke subjects [29,31,32]. During stroke, glutamate is released in supraphysiological quantities that cause neurotoxicity within the brain [33]. Phenylalanine exerts a selective, significant, and reversible depression of ionotropic glutamate receptor function at excitatory synapses in hippocampal and cerebrocortical neuronal cultures prepared from rats or mice via a unique set of presynaptic and postsynaptic mechanisms in stroke. Therefore, phenylalanine increase is regarded to be a compensatory response to supraphysiological and neurotoxic quantities of glutamate [29,34].

Network analysis revealed that GSK3B and NOS2 are strongly associated with the metabolites highlighted in the metabolomics analysis, indicating that these two targets are probably the potential target for the protective effect of GSTTF against the MCAO. Inducible nitric oxide synthase produces nitric oxide (NO) which is a messenger molecule with diverse functions throughout the body. Oxygen-free radicals and oxidants appear to play an essential role in central nervous system injury after cerebral ischemia and reperfusion. The excessive production of NO with the further formation of the toxic compound peroxynitrite anion ONOO^–^, is currently considered to be one of the most important mechanisms of tissue damage at various stages of brain ischemia [35,36,37]. The quantity of NO synthesized by NOS2 is thousands of times higher than the total quantity of NO formed by NO synthase. The expression of NOS2 in the cells of ischemic tissue and the surrounding potentially viable zone has been considered to be one of the factors that determine the distribution of tissue damage at the later stages of cerebral ischemia [37]. 

Glycogen synthase kinase-3 (GSK3) is a serine/threonine kinase comprising two distinct isoforms, GSK3A and GSK3B, which is highly enriched in the mammalian brain and involved in diverse cellular and neurophysiological functions [38,39]. Glycogen synthase kinase-3 serves as a regulator of apoptosis and inflammation, known contributors to stroke-induced cell death. Dysregulation of GSK3-mediated substrate phosphorylation and signaling pathways has been confirmed to be associated with the pathophysiological conditions of a variety of diseases, including Alzheimer’s disease, type 2 diabetes, and cancer [38]. Glycogen synthase kinase-3 inhibition has attracted extensive attention as one of the critical therapeutic targets, and GSK3B has been deeply involved in the neuronal cell death caused by cerebral ischemic insult [40]. 

## 4. Material and Methods

### 4.1. Materials

Chromatographic grade methanol, acetonitrile, and formic acid were purchased from Fisher Scientific (FairLawn, NJ, USA). Ultrapure water was prepared using a Milli-Q purification system (Billerica, MA, USA). The gross saponins of *Tribulus terrestris* fruit (purity of saponins: above 60%, Lot No. 0418303) was manufactured by Changbaishan Pharmaceutical Co. Ltd. (Jilin, China). 

### 4.2. Animals and Treatments

A total of 30 adult male Sprague–Dawley rats weighing 200 ± 20 g was purchased from the Beijing Vital River Laboratory Animal Technology Co., Ltd. (Beijing, China). Rats were housed in a climate-controlled room with a 12 h light–dark illumination cycle at 40%–65% relative humidity and 19–23 °C. The whole animal treatments were approved by the Animal Ethics Committee, Academy of Traditional Chinese Medicine of Jilin Province (approval No. JLSZKYDWLL-2018-015). 

After two weeks acclimatization, a total of 30 rats were divided randomly into three groups, including the sham group, MCAO group, and GSTTF-treated group. The administration of GSTTF (3 mg/kg) was performed via tail vein injection for three days before and 24 h after MCAO surgery for GSTTF-treated rats. The sham-operated and MCAO rats were administrated with the same volume of saline. 

All rats were anesthetized with 10% chloral hydrate at a dose of 3 mL/kg body weight (i.p.) before surgery. The MCAO surgery was performed following the previously reported with modifications [41,42]. Briefly, a 1 cm long midline skin incision was made on the neck; then, the muscle on either side of the trachea was separated to expose the common carotid artery (CCA), the internal carotid artery (ICA), and the external carotid artery (ECA). Then a silicone-coated suture was inserted from the left ECA into the lumen of the ICA to occlude the origin of the MCA. The rats in the sham-operated group were subjected to an identical procedure without the ligation. All animals were maintained at 25–28 °C during the surgery.

The 24 h urine samples of rats were collected into tubes, then were centrifuged at 3000× *g* at 4 °C for 10 min and were frozen immediately at −80 °C until metabolomics analysis. The brain samples were stored at −80 °C. 

### 4.3. Infarct Volume Measurement

The brains were removed and sliced into 2 mm thick coronal sections and stained with 2,3,5-triphenyltetrazolium chloride (TTC, 2% TTC in phosphate-buffered saline). All coronal slices were digitalized, and the area of cerebral damage was analyzed using Image-J software (National Institutes of Health, Bethesda, MD, USA).

### 4.4. Evaluation of Neurological Defects

Neurological functional deficiency scores were in accordance with Longa’s five-point scale [13,42]: zero points—no neurobehavioral dysfunction; one point—failure to flex the contralateral front limb completely; two points—circling counter clockwise; three points—turning around to the affected side seriously; four points—cannot walk spontaneously. The higher the score, the more serious the impairment of animal behavior.

### 4.5. Urine Sample Preparation

All stored frozen urine samples were thawed on the ice and were mixed before use. A 200 μL urine sample was centrifuged at 10,000× *g* for 10 min at 4 °C to remove particulates, and 100 μL of supernatant were transferred to another tube. It was then added to 100 μL water and mixed well for LC-MS analysis [43]. A pooled “quality control” (QC) sample was prepared by mixing an equal aliquot (20 μL) from all urine samples and prepared using the above method for the optimization of the chromatographic and MS conditions and method validation. 

### 4.6. LC-MS Analysis

Chromatographic separation was performed with a Vanquish Duo UHPLC system (Thermo Fisher Scientific, San Jose, CA, USA) using a Hypersil GOLD^TM^ column (2.1 mm × 50 mm, 1.9 µm) with a column temperature at 50 °C and an injection volume of 5 μL. The mobile phase consisted of 0.1% aqueous formic acid (phase A) and acetonitrile containing 0.1% formic acid (phase B) under the following gradient program: 0.5% B (0–1 min); 0.5–1% B (1–2 min); 1–5% B (2–3 min); 5–15% B (3–8 min); 15–50% B (8–10 min); 50–95% B from (10–12 min); and 95–0.5% B (12–13 min) and maintained at 0.5% for five minutes. The whole duration was 18 min at a flow rate of 0.4 mL/min, and all samples were maintained at 4 °C through the analysis.

The mass acquisition was conducted on a Q-Orbitrap mass spectrometer equipped with an electrospray ionization (ESI) source (Thermo Fisher Scientific, San Jose, CA, USA) operating separately in both negative and positive ion modes. The mass resolution was set as 35,000 for full-scan analysis with a mass range of *m*/*z* 100–1500, while the resolution reduced to 17,500 for the data-dependent (Top 5) acquisition to obtain the tandem mass spectrum. The ESI source parameters were as follows: capillary voltage of 3.5 kV in positive ion mode or −3.2 kV in negative ion mode, sheath gas flow 50 arb, auxiliary gas flow 15 arb, sweep gas flow 2 arb, and capillary temperature 300 °C. Prior to data acquisition, the mass spectrometer was calibrated using Pierce™ negative and positive ion calibration solution (Thermo Fisher Scientific, San Jose, CA, USA). 

### 4.7. Data Analysis and Biomarker Selection

The raw UHPLC-Q-Orbitrap/MS data of urine samples were processed by the Compound Discoverer (CD version 3.0 Thermo Scientific) which incorporates a peak deconvolution package to detect the mass, retention time, and intensity of the peaks in each chromatogram. After being extracted and aligned, the intensity of each ion was normalized by the total ion intensity of each chromatogram. The resultant dataset, containing the *m*/*z* values, the normalized intensity (variables), and the sample code (observations), was exported as a. csv file and imported into SIMCA software package (version 13.0, Umetrics, Umeå, Sweden) to conduct multivariate statistical analysis.

Partial least squares discriminant analysis (PLS-DA) was applied to exhibit the metabolite differences among different groups. The parameters R2 and Q2 (cum) were used to evaluate the quality of the model. Candidate biomarkers were selected from the comparison between the sham group and model group by the combination of the variable importance in the projection (VIP) values (VIP > 2) and fold change values (fold change > 2). Then the Student’s *t*-test was further used to assess the significant differences of these metabolites among groups using GraphPad Prism 6.0 (La Jolla, CA, USA). The metabolites with the value of *p* < 0.05 were considered statistically significant and selected as a biomarker. Then the intensity changes of these biomarkers between the GSTTF-treated group and the model group were calculated to evaluate the effect of GSTTF for MCAO.

The annotation of urine metabolite was accomplished by matching the accurate mass and MS/MS data of endogenous mammalian metabolites acquired from available databases, such as the Human Metabolome Database (HMDB) and METLIN. The metabolic pathway analysis of potential biomarkers was implemented using MetaboAnalyst 4.0 (McGill University, Montreal, QC, Canada).

### 4.8. Network Pharmacology Analysis

The Traditional Chinese Medicine Systems Pharmacology Database and Analysis Platform (TCMSP) is a unique systems pharmacology platform for Chinese herbal medicines that captures the relationships among drugs, targets, and diseases [44]. The active component of *Tribulus terrestris* as well as the literature-supported compound targets and the encephalopathy-related targets were obtained from the TCMSP database. 

The procedure for network construction was as follows: (1) The ‘‘active component-candidate target (C-C) network’’ was constructed by linking the candidate compounds and all their candidate targets, and (2) the ‘‘disease–target (D-T) network’’ was established by connecting the encephalopathy-related diseases and therapeutic targets. All networks were visualized and analyzed using Cytoscape 3.7.0, in which the molecular species (compounds and proteins) or diseases were represented as nodes and intermolecular interactions (compound–target or target–disease interactions) were indicated as links, i.e., edges among nodes [45]. The disease-related targets that can also be modulated by the active compounds were selected, and then the correlations between these targets and the metabolites highlighted from the metabolomics analysis were analyzed using the IMPaLA web tool. Finally, the targets that could connect disease, active compounds, and metabolites were selected as the potential therapeutic targets.

## 5. Conclusions

An LC-MS-based metabolomics approach combined with network pharmacology analysis was employed to study the protective effect of GSTTF against MCAO-induced ischemic stroke. A set of 14 metabolites were annotated as biomarkers, in which 13 of them could be reversed by GSTTF, and the metabolic pathway analysis revealed that the protective effect of GSTTF was probably related to the regulation of BCAAs, and that citric acid and phenylalanine were impacted by MCAO surgery. The network analysis screened two targets, NOS2 and GSK3B, which was probably related to the protective effect of GSTTF. These changes in metabolites and targets, as well as the metabolite–target pathway network, could provide insights into the mechanism of MCAO-induced ischemic stroke. However, in-depth investigations are needed, which will be beneficial to understanding the regulation mechanism more clearly, and further allow for the development of GSTTF as a potential complementary therapeutic drug for ischemic stroke treatment in the future.

## Figures and Tables

**Table 1:** Annotated endogenous metabolites and their change trends in urine.

Annotated Metabolite	Ionization Mode	Retention Time (min)	Detected *m*/*z*	Adducts	KEGG ID	Model/Sham	GSTTF/Model
Leucine	P	0.708	132.1017	[M + H]^+^	C00123	↑^#^	↓
Citric acid	P	0.527	193.0339	[M + H]^+^	C00158	↓^#^	↑
Citric acid	N	0.497	191.0190	[M − H]^−^	C00158	↓^#^	↑**
Isoleucine	P	0.659	132.1016	[M + H]^+^	C00407	↓^#^	↑
d-Pantothenic acid	P	2.928	220.1174	[M + H]^+^	C00864	↑^#^	↓
Hippuric acid	P	4.044	180.0653	[M + H]^+^	C01586	↓^##^	↑*
Hippuric acid	N	3.999	178.0502	[M − H]^−^	C01586	↓^#^	↑
l-Pyroglutamic acid	P	0.571	130.0497	[M + H]^+^	C01879	↓^###^	↑
Citraconic acid	N	0.416	129.0182	[M − H]^−^	C02226	↓^##^	↑
trans-Aconitic acid	N	0.591	173.0083	[M − H]^−^	C02341	↓^##^	↓
N-Acetyl-l-leucine	P	5.387	174.1122	[M + H]^+^	C02710	↑^##^	↓
N-Acetyl-l-leucine	N	6.020	172.0972	[M − H]^−^	C02710	↑^##^	↓**
3-Hydroxy-3-methylglutaric acid	N	0.525	161.0447	[M − H]^−^	C03761	↑^##^	↓
6-Hydroxycaproic acid	N	4.550	131.0704	[M − H]^−^	C06103	↑^###^	↓
N-Acetyl-l-Citrulline	N	0.531	216.0986	[M − H]^−^	C15532	↑^###^	↓**
7-Aminomethyl-7-deazaguanine	P	1.322	180.0876	[M + H]^+^	C16675	↑^#^	↓
Pentahomomethionine	P	9.059	220.1361	[M + H]^+^	C17229	↑^##^	↓

^#^*p* < 0.05, ^##^ *p* < 0.01, ^###^ *p* < 0.001, the model group versus the sham group; * *p* < 0.05, ** *p* < 0.01, *** *p* < 0.001, the GSTTF-treated group versus the model group. P, positive ion mode; N, negative ion mode.

## Data Availability

The data from this study are available from the authors upon request.

## Supplementary Materials

The following are available online at https://www.mdpi.com/2218-1989/9/10/240/s1, Figure S1: Representative base, peak chromatograms of urine samples from different samples acquired in both positive (A–C) and negative ion modes (D–E); Figure S2: PLS-DA permutation test of the sham and model groups based on the data obtained in positive ion mode (A) and negative ion mode (B); PLS-DA permutation test of the sham, model, and GSTTF-treated groups based on the data obtained in the positive ion mode (C) and negative ion mode (D); Figure S3: Mass spectra of the hippuric acid ion at RT 3.99_*m*/*z* 178.0502: (A) full-scan mass spectrum, (B) tandem mass spectrum; Figure S4 The compound–target network (A) and disease–target network (B); Table S1, Information on the targets highlighted in this study.
