# Supplementary material for: Investigating the Protective Effect of Gross Saponins of Tribulus terrestris Fruit against Ischemic Stroke in Rat Using Metabolomics and Network Pharmacology
Source: Metabolites. 2019 Oct 21;9(10):240. doi: 10.3390/metabo9100240 (PMC6835270; doi:10.3390/metabo9100240)
Supplement: Supplementary file 1 [file metabolites-09-00240-s001.zip › metabolites-604289 - Supplementary material.pdf]

Figure S1

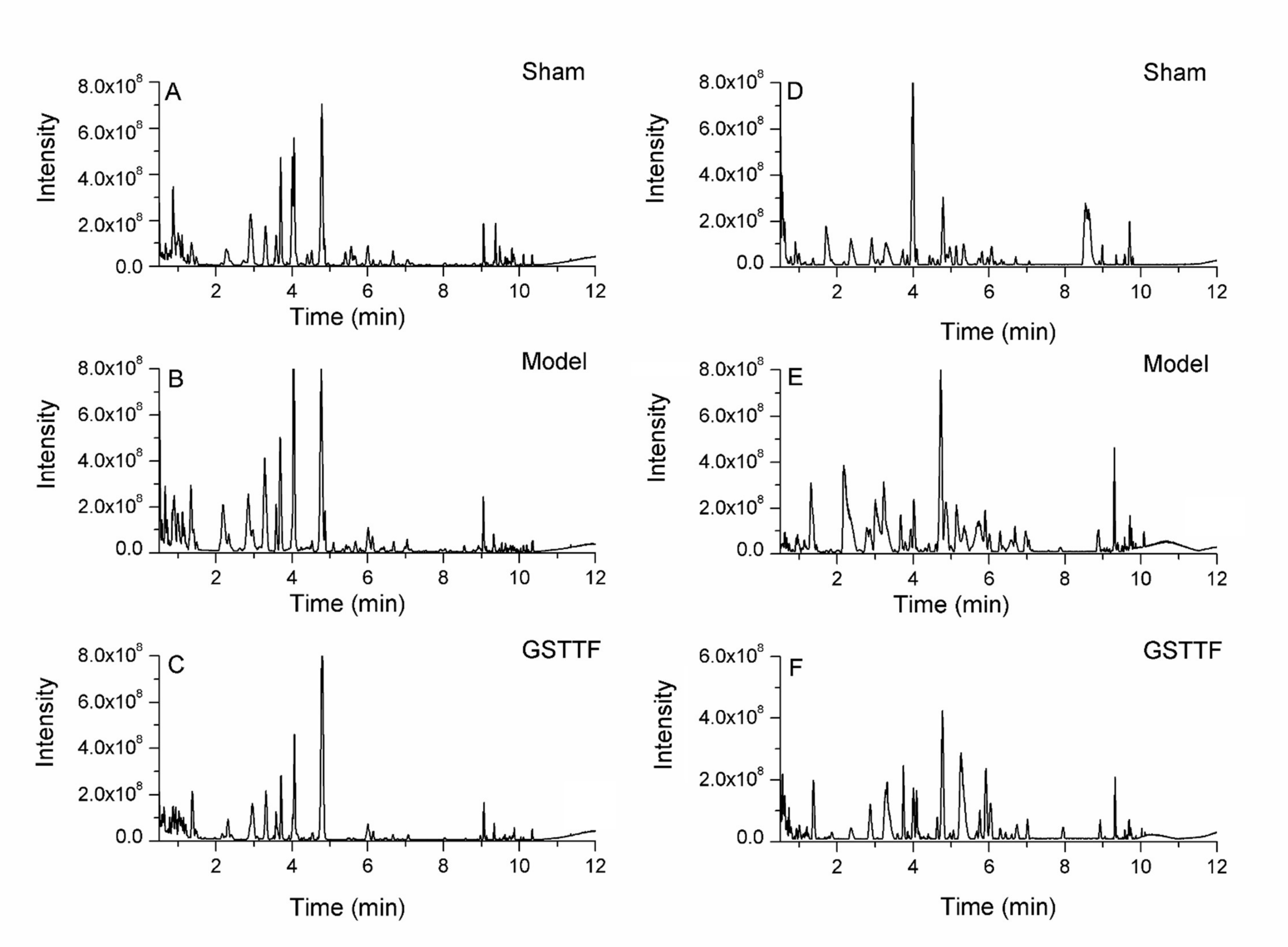

Figure S1 Representative base peak chromatograms of urine samples from different samples acquired in both positive (A-C) and negative ion modes (D-E).

Figure S2

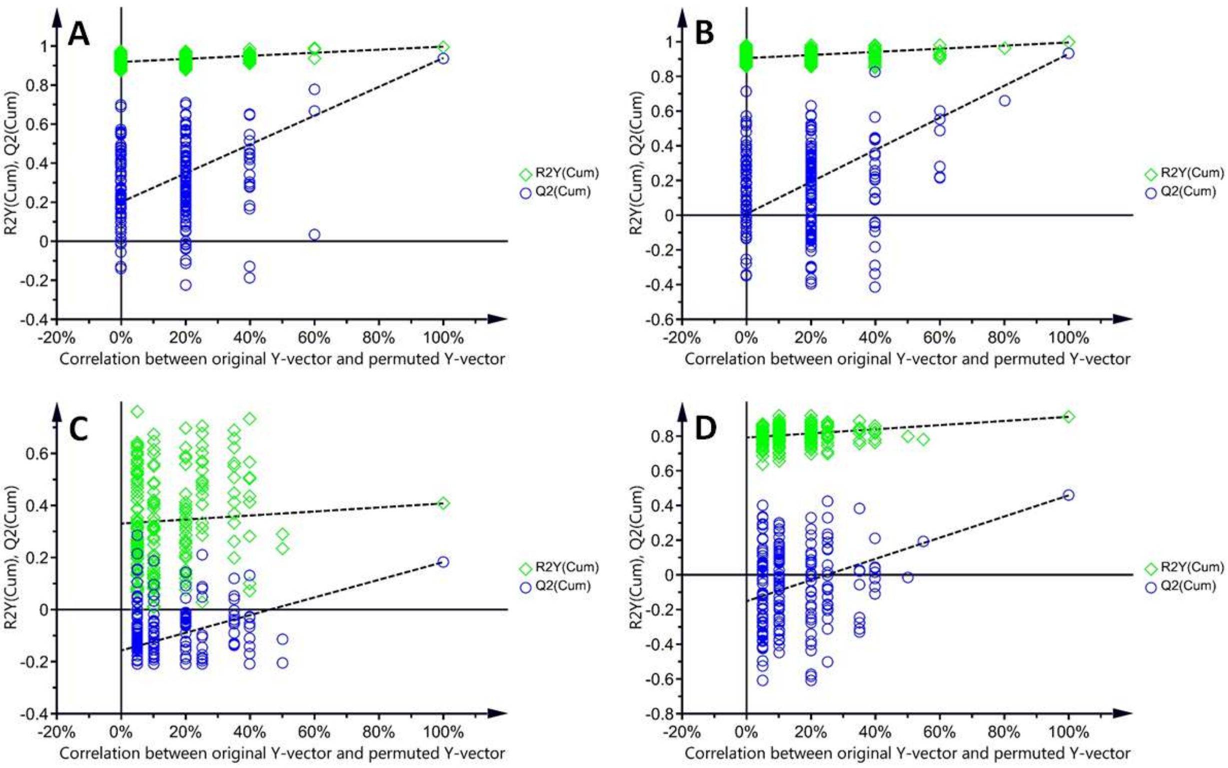

Figure S2 PLS-DA permutation test of Sham and model groups based on the data obtained in positive ion mode (A) and negative ion mode (B); PLS-DA permutation test of Sham, model and GSTTF-treated groups based on the data obtained in positive ion mode (C) and negative ion mode (D).

Figure S3

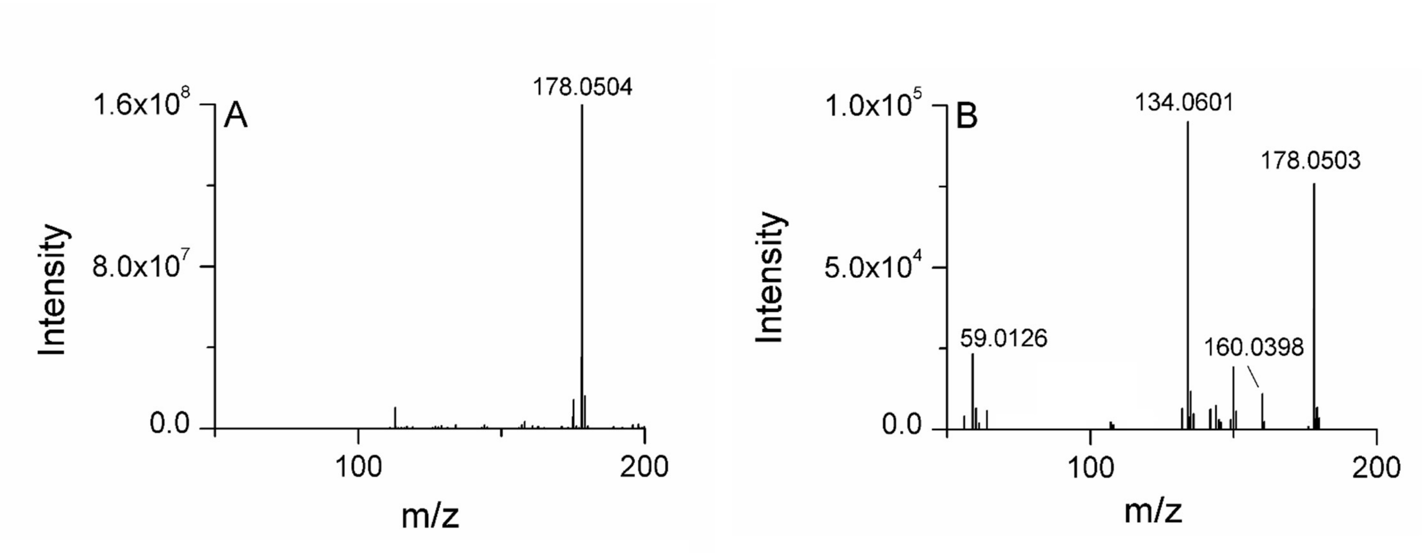

Figure S3 Mass spectra of the hippuric acid ion at RT 3.99_m/z 178.0502, (A) full scan mass spectrum, (B) tandem mass spectrum.

Figure 4

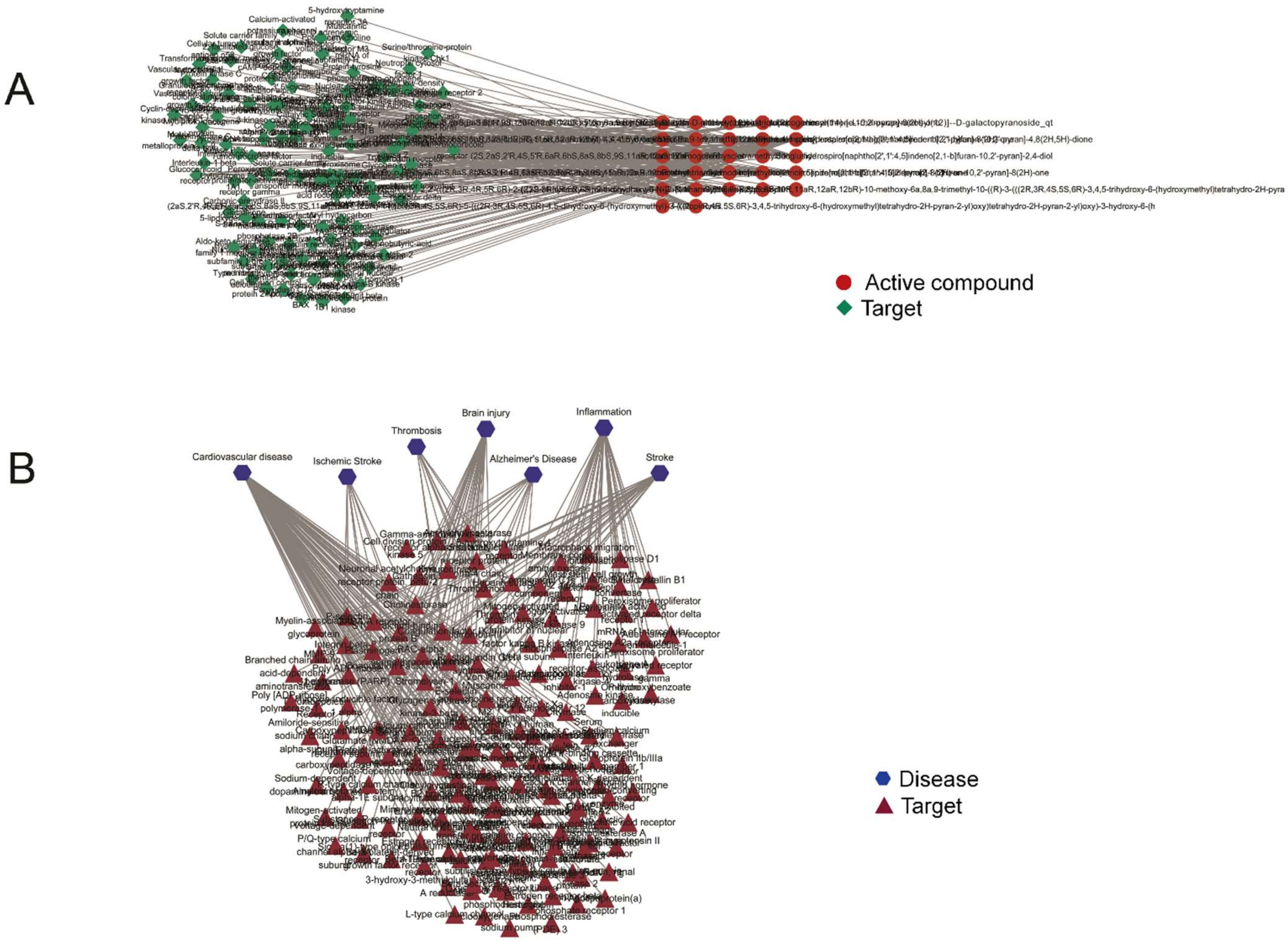

Figure S4 The compound-target network (A) and disease-target network (B).
