# Supplementary material for: Investigating the Protective Effect of Gross Saponins of Tribulus terrestris Fruit against Ischemic Stroke in Rat Using Metabolomics and Network Pharmacology
Source: Metabolites. 2019 Oct 21;9(10):240. doi: 10.3390/metabo9100240 (PMC6835270; doi:10.3390/metabo9100240)
Supplement: Supplementary file 1 [file metabolites-09-00240-s001.zip › metabolites-604289 - Supplementary Table S1.pdf]

Table S1 The information of the targets highlighted in this study

Target name	Gene symbol
Acetylcholinesterase	ACHE
Androgen receptor	AR
Beta-2 adrenergic receptor	ADRB2
Cell division protein kinase 2	CDK2
CGMP-inhibited 3',5'-cyclic phosphodiesterase A	PDE3A
Coagulation factor Xa	F10
E-selectin	SELE
Estrogen receptor	ESR1
Estrogen receptor beta	ESR2
Glycogen synthase kinase-3 beta	GSK3B
Interleukin-1 beta	IL1B
Leukotriene A-4 hydrolase	LTA4H
Mineralocorticoid receptor	NR3C2
Mitogen-activated protein kinase 14	MAPK14
Mitogen-activated protein kinase 8	MAPK8
Muscarinic acetylcholine receptor M1	CHRM1
Muscarinic acetylcholine receptor M2	CHRM2
Nitric oxide synthase, inducible	NOS2
Peroxisome proliferator activated receptor delta	PPARD
Peroxisome proliferator activated receptor gamma	PPARG
Prostaglandin G/H synthase 1	PTGS1
Prostaglandin G/H synthase 2	PTGS2
